# Influence of Mg Doping Levels on the Sensing Properties of SnO_2_ Films

**DOI:** 10.3390/s20072158

**Published:** 2020-04-10

**Authors:** Bouteina Bendahmane, Milena Tomić, Nour El Houda Touidjen, Isabel Gràcia, Stella Vallejos, Farida Mansour

**Affiliations:** 1 Electronic Materials Study for Medical Applications (LEMEAMED) Laboratory, Electronic Department, Science and Technology Faculty, Frères Mentouri University, 25000 Constantine, Algeria; houdatouidjen@yahoo.fr (N.E.H.T.); farida.mansour@yahoo.fr (F.M.); 2 Instituto de Microelectrónica de Barcelona (IMB-CNM, CSIC), Campus UAB, 08193 Bellaterra, Spain; milena.tomic@imb-cnm.csic.es (M.T.); isabel.gracia@imb-cnm.csic.es (I.G.); 3 CEITEC-Central European Institute of Technology, Brno University of Technology, 61200 Brno, Czech Republic

**Keywords:** Mg-doped SnO_2_, spray pyrolysis, thin films, gas sensing, volatile organic compounds

## Abstract

This work presents the effect of magnesium (Mg) doping on the sensing properties of tin dioxide (SnO_2_) thin films. Mg-doped SnO_2_ films were prepared via a spray pyrolysis method using three doping concentrations (0.8 at.%, 1.2 at.%, and 1.6 at.%) and the sensing responses were obtained at a comparatively low operating temperature (160 °C) compared to other gas sensitive materials in the literature. The morphological, structural and chemical composition analysis of the doped films show local lattice disorders and a proportional decrease in the average crystallite size as the Mg-doping level increases. These results also indicate an excess of Mg (in the samples prepared with 1.6 at.% of magnesium) which causes the formation of a secondary magnesium oxide phase. The films are tested towards three volatile organic compounds (VOCs), including ethanol, acetone, and toluene. The gas sensing tests show an enhancement of the sensing properties to these vapors as the Mg-doping level rises. This improvement is particularly observed for ethanol and, thus, the gas sensing analysis is focused on this analyte. Results to 80 ppm of ethanol, for instance, show that the response of the 1.6 at.% Mg-doped SnO_2_ film is four times higher and 90 s faster than that of the 0.8 at.% Mg-doped SnO_2_ film. This enhancement is attributed to the Mg-incorporation into the SnO_2_ cell and to the formation of MgO within the film. These two factors maximize the electrical resistance change in the gas adsorption stage, and thus, raise ethanol sensitivity.

## 1. Introduction

The monitoring of volatile organic compounds (VOCs), including ethanol (C_2_H_6_O), acetone (C_3_H_6_O), and toluene (C_7_H_8_), is routinely needed for evaluating environmental quality and industrial safety [1]. Recently, the monitoring of VOCs has also gained importance in clinical applications as a promising tool to identify pathological conditions at early stages (via breath analysis of anomalous concentrations of certain VOCs) [2]. In this context, metal oxides (MOXs)-based gas sensors (chemoresistive sensors) are attractive devices that can be miniaturized and integrated as array systems into compact VOC monitoring equipment at reduced fabrication costs as compared to other technologies (e.g., spectrometers) [1,2]. Previously, MOXs such as ZnO, SnO_2_, WO_3_, and TiO_2_ at the nanoscale have proved promising properties to sense VOCs due to their high surface-to-volume ratio, which provides better sensitivity and stability compared to bulk MOXs [3,4,5,6,7]. Currently, however, these materials require yet the enhancement of sensitivity to low concentrations of VOCs and, generally, the improvement of selectivity and the minimization of drift effect over time. Thus, various efforts have been focused on this line, pointing out the importance of enhancing both chemical and electronic sensitization by incorporating intentional impurities (e.g., doping) or modifying the surface of traditional gas-sensitive MOXs [6,8].

Tin dioxide (SnO_2_) is considered as one of the leading n-type MOXs used in commercial chemoresistive gas sensors, however, the enhancement of its sensing performance remains active to date [9,10]. As mentioned above for other MOXs, a large series of reports has also emphasized the remarkable improvement of SnO_2_ sensing characteristics, whether by adding catalysts, introducing functional activators, or doping with impurities [5,10,11]. The latter has proved to be an efficient way to create more oxygen vacancies and to amplify the electrochemical reaction of analytes on the layer’s surface [7,12].

In the literature, there are several examples of noble metals (Pd, Pt), rare earth metals (Ce, Pr), and metals (Zn, Al) improving the sensitivity and selectivity of various MOXs including SnO_2_ [10,12,13,14,15,16]. Among these materials, magnesium (Mg) is attractive, as it has proved to improve sensitivity to ethanol, H_2_, CO, and ammonia when incorporated into ZnO to form Mg-doped ZnO [17,18,19,20,21]. Similarly, Mg-doped TiO_2_ and Mg-doped In_2_O_3_ have shown to improve sensitivity to CO and ethanol, respectively [3,22]. Mg is also considered a promising candidate to enhance SnO_2_ sensing properties, particularly as it presents a close ionic radius (0.67 nm) to that of Sn (0.71 nm), which facilitates Mg^2+^ diffusion into the SnO_2_ cell to substitute Sn^4+^ [23]. Despite this, the use of Mg-doped SnO_2_ in gas sensing, particularly VOC sensing, is not that common in the state-of-the-art. 

In the midst of various techniques used to synthesize doped MOX films, spray pyrolysis proved to be simple, cost effective and allowed a wide variety of substrate coatings [24]. Despite the fact that controlling the size and rate of sprayed droplets remains a challenging factor for films’ quality, spray pyrolysis is considered a useful method for the design and development of chemoresistive gas sensors [24,25]. 

Previously, we proved the spray pyrolysis deposition of SnO_2_ films and their potential to detect vapors such as ethanol, methanol and acetone [26]. Herein, we go further into this line and investigate the influence of Mg doping levels on the VOC sensing properties of SnO_2_ thin films synthesized via the spray pyrolysis process. This work explores the structural, morphological, and chemical composition properties of the films and their gas sensing properties towards ethanol, acetone and toluene.

## 2. Materials and Methods

### 2.1. Thin Film Synthesis and Processing

Magnesium-doped tin dioxide thin films with three doping concentrations (0.8, 1.2, and 1.6 at.%) were synthesized by spray pyrolysis method on cleaned amorphous glass substrate (70 × 30 × 1 mm) at 400 °C. To obtain a 0.8 at.% Mg-doped SnO_2_ film, a mixture of 1088 mg of tin (II) chloride dihydrate (SnCl_2_·2H_2_O, Sigma-Aldrich, 98%), 40 mg of magnesium chloride hexahydrate (MgCl_2_-6H_2_O, Sigma-Aldrich, 99%), and 100 mL of ethanol (Sigma-Aldrich, 96%) was stirred under heat for 30 min. Afterwards, this solution was sprayed by a nozzle to the heated substrate using airflow (0.5 mL/min) for 30 min. The same process was used to prepare all films, varying the weight ratio of the precursors according to the doping concentrations. The as-prepared Mg-doped SnO_2_ thin films were subsequently annealed at 450 °C in dry air for 60 min in order to ensure the stability of the materials during the gas sensing test. The influence of the annealing temperatures on the SnO_2_ thin films’ properties was reported in our previous work [27]. Finally, the coated samples were cut into parts (10 × 15 × 1 mm) to be used for characterization and gas tests.

### 2.2. Characterization Methods

Structural characterization was performed using an X-ray Diffractometer (XRD—Bruker-AXS, LinxEye XE-T detector, KFL Cu 2K (λ (CuKα) = 1.541840 Å) operated at 40 KV and 40 mA) in the 2*θ* angle from 20° to 80°. XPowder software was employed to refine (peak position, lattice parameters, and crystallites size) the data. The surface morphology was examined by Scanning Electron Microscopy (SEM- Carl Zeiss, Auriga Series) equipped with an EDX spectrometer (Energy Dispersive X-ray Spectroscopy) for elemental composition analysis. The surface properties of the films were analyzed using X-ray Photoelectron Spectroscopy (XPS—Kratos Axis Supra with monochromatic Al Kα X-ray radiation, emission current of 15 mA and hybrid lens mode, Manchester, UK) varying the binding energy (BE) range between 0−1350 eV and using CasaXPS for peaks fitting.

### 2.3. Gas Sensing Measurements

**Figure 1:**
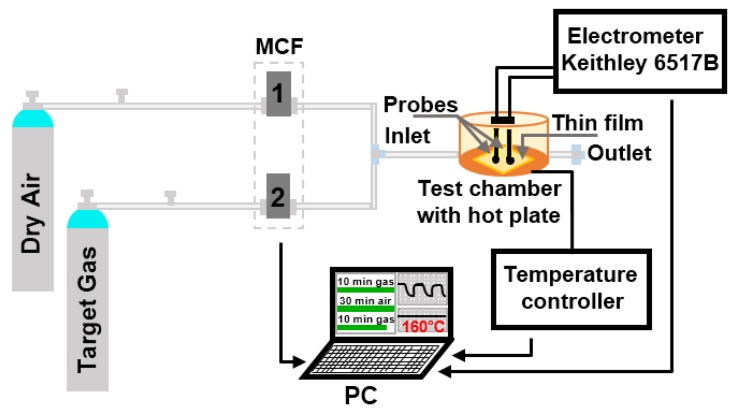
Schematic view of the gas sensing measurement system.

To evaluate the gas sensing properties of Mg-doped SnO_2_ thin films towards various gaseous analytes (ethanol, acetone, and toluene), we used the system represented in Figure 1 and reported previously [28]. Briefly, the system consists of a continuous gas supply (dry synthetic air and different calibrated gas analytes) controlled by mass flow controllers (MFC), a test chamber equipped with two probes as the electrical signal collectors, a heating plate with a temperature controller, and a data acquisition system (electronic measurement system, electrometer, and PC). The films were exposed to each analyte for 10 min, and after that, the chamber was purged with synthetic air for 30 min to recover the initial base line resistance. Gas test measurements were recorded fixing the operating temperature (T_op_) to 160 °C and varying the analyte concentration from 10 to 80 ppm. The gas response is defined as R_a_/R_g_ (reducing gases), where R_a_ and R_g_ are the resistances of the film in dry air and after exposure to analyte, respectively, while sensitivity (S), is the report between response and gas concentration (∆R/∆C) [7]. The response and recovery times are defined as the time required for the sensor to reach 90% of the response upon exposure to the target gas, and the time required for a sensor to return to 90% of the original baseline signal upon removal of the target gas, respectively. All samples were alternatively tested towards the target analytes in dry air accumulating a total operating period of 120 h distributed over 30 days. Among these hours, on average, each sample operated for about 20 h. Hereafter, the tested films are labeled as MTO1 (0.8 at.% magnesium-doped tin dioxide), MTO2 (1.2 at.% magnesium-doped tin dioxide), and MTO3 (1.6 at.% magnesium-doped tin dioxide).

## 3. Results and Discussion

### 3.1. Films Analysis and Characterization

**Figure 2:**
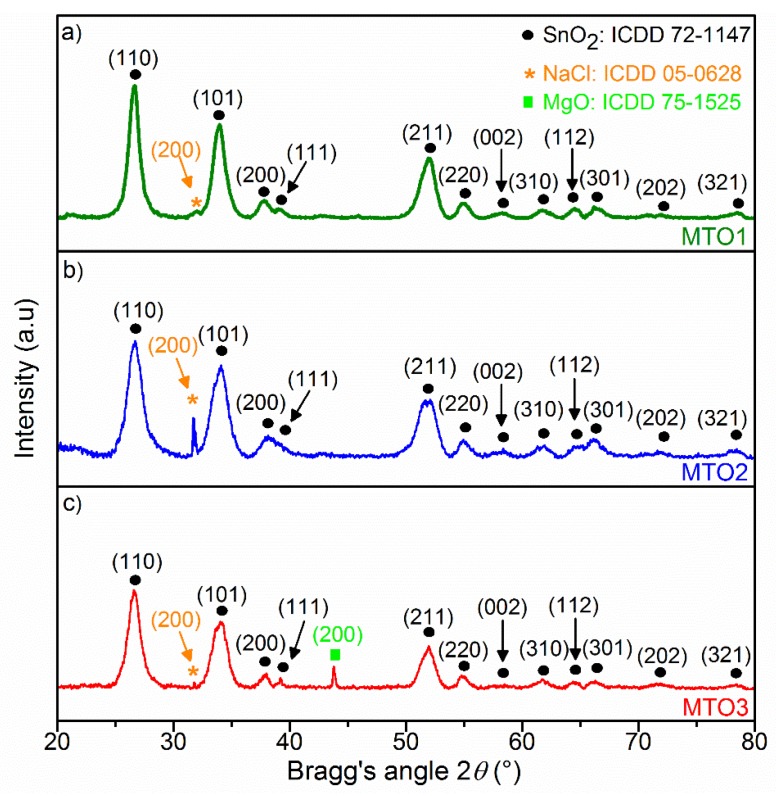
XRD patterns of the Mg-doped SnO_2_ thin films, (**a**) 0.8 at.% magnesium-doped tin dioxide (MTO1); (**b**) 1.2 at.% magnesium-doped tin dioxide (MTO2); (**c**) 1.6 at.% magnesium-doped tin dioxide (MTO3).

X-ray diffraction patterns were evaluated to gain insight into the crystal structure of the Mg-doped SnO_2_ thin films. As exhibited in Figure 2, all films are polycrystalline in nature and crystallize in rutile tetragonal tin dioxide (SnO_2_) structure (P4_2_/mnm space group, ICDD card 72−1147) with a dominant (110) diffraction at 26.53° 2*θ* approximately. In general, the results show a steady decrease of all SnO_2_ diffraction peaks accompanied by a diffraction pattern shift to lower angle values (>0.06° 2*θ*) as the Mg amount increases in the films. This could be caused in part by the diffusion of Mg^2+^ ions into the SnO_2_ lattice and the smaller ionic radii of the guest atom (Mg^2+^: 0.67 Å) compared to the host atom (Sn^4+^: 0.71 Å), and also by the lower electronic density of Mg atoms (1.738 g/cm^3^) compared to Sn atoms (β−Sn = 7.265 g/cm^3^). The results also indicate a change in the lattice parameters of the Mg-doped SnO_2_ thin films (Table 1), which reveal a slight increase in “*a*” and decrease in “*c*”, compared to the non-doped SnO_2_ film reported in our previous work [27]. In addition, a proportional decrease of the average crystallite size (D) with the increase of the Mg-doping levels can also be observed in Table 1. Overall, these properties suggest the presence of local lattice disorders in the films and, in turn, an increment of surface defects in the Mg-doped samples with respect to the non-doped samples, as noticed earlier in the literature [29]. 

The ‘extra’ diffraction peak at 43.8° 2*θ* in the MTO3 pattern is associated with the (200) reflection of MgO in halite structure (ICDD 75−1525). The presence of this diffraction suggests an excess of Mg (over-doping) in MTO3 and the formation of a secondary phase. This is consistent with the unit cell parameters found for MTO3 (Table 1), which show no further change with respect to MTO2, and endorse the fact that the substitution process goes to its saturation. These observations are in agreement with previous reports on Mg-doped SnO_2_ by the sol-gel process [30]. Moreover, the Mg-doped SnO_2_ patterns also revealed additional diffraction peaks referring to the formation of NaCl crystals (ICDD 05−0628) on the matrix of the films. This could be attributed to the combination of sodium ions (diffused from the glass substrate) and chloride ions (from the metal chloride precursor) to form NaCl crystals [31].

**Figure 3:**
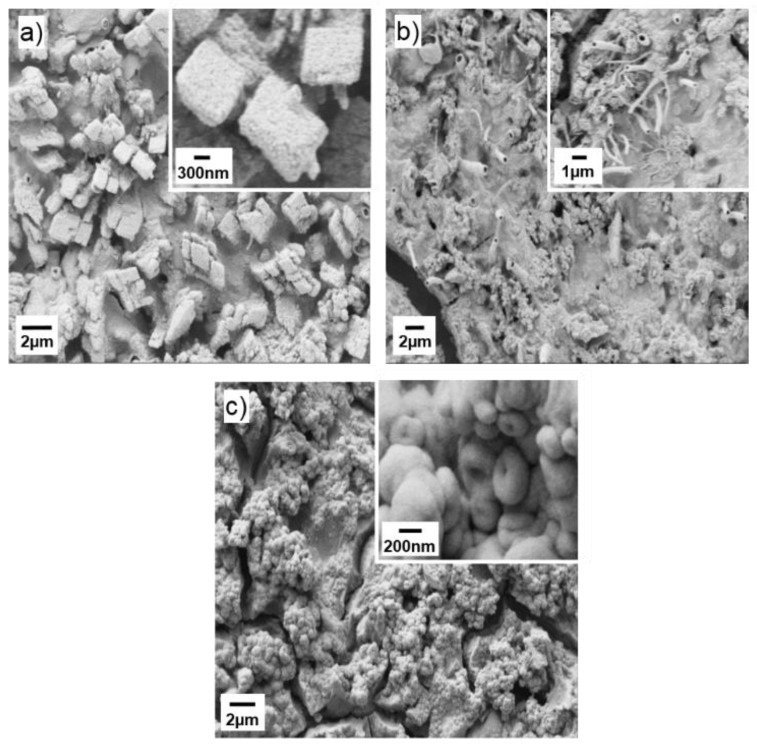
SEM micrographs of the Mg-doped SnO_2_ thin films, (**a**) MTO1; (**b**) MTO2; (**c**) MTO3.

SEM micrographs of the Mg-doped SnO_2_ thin films (Figure 3) display different morphologies with relatively rough topography and irregular particle distribution in all samples. Low magnification SEM images of the films also reveal cracked patterned surfaces after the annealing treatment. Specifically, the SEM images for the MTO1 films (Figure 3a) display the agglomeration of the nanoparticles in cubic shape, whereas the MTO2 films (Figure 3b) show the presence of several hollow microtubes distributed randomly on the surface, and the MTO3 films (Figure 3c) exhibit agglomeration of nanospheres.

EDS analysis corroborated the presence of the three main elements in the films Sn, O, and Mg, with proportional increase of Mg atomic percentage according to the doping level. The presence of other elements, such as Si and Na (associated with the use of glass substrate), and Cl (related to the chloride-based precursors), are also registered and in line with the XRD results. Further properties of the elements at the surface of the films were investigated by XPS.

**Figure 4:**
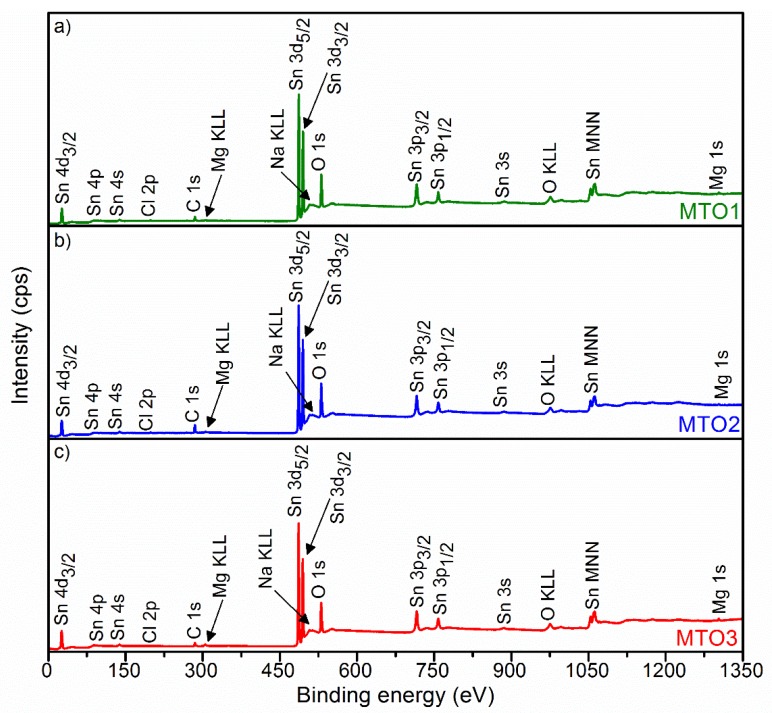
XPS survey spectra of the Mg-doped SnO_2_ thin films, (**a**) MTO1; (**b**) MTO2; (**c**) MTO3.

Figure 4 displays the survey XPS spectrum for each film (i.e., MTO1, MTO2 and MTO3). These spectra show a sharp and intense XPS core level Sn 3d and O 1s peaks around 486.5 eV and 530.3 eV, respectively. The spectra also show minor peaks for Sn 4d_3/2_, Sn 4p, Sn 4s, C 1s, Mg KLL, Sn 3p_3/2_, Sn 3p_1/2_, Sn 3s, O KLL, Sn MNN, and Mg 1s. Na KLL and Cl 2p peaks are also identified in the spectra in concordance with the XRD and EDS results. 

**Figure 5:**
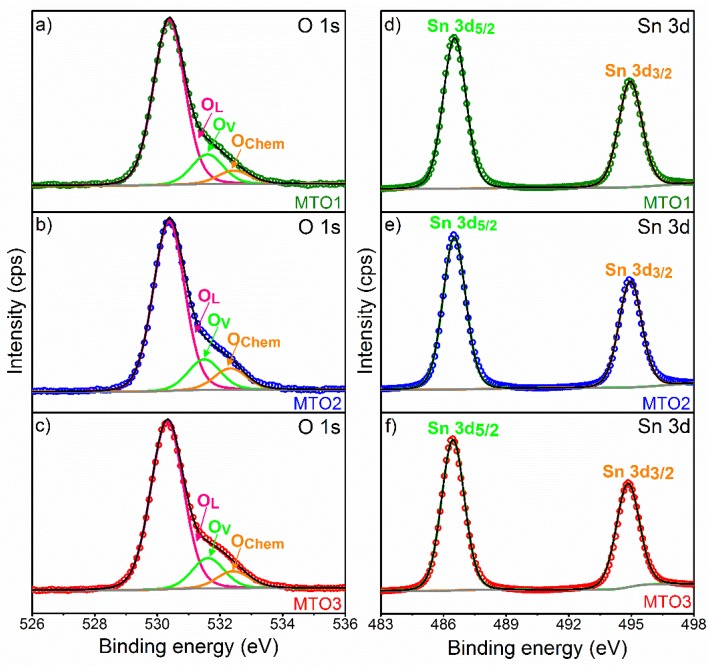
Deconvolution of O 1s (**left figure**) and Sn 3d (**right figure**) XPS core level spectra, ((**a**) and (**d**)) MTO1; ((**b**) and (**e**)) MTO2; ((**c**) and (**f**)) MTO3. The circles denote experimental data, colored lines demonstrate the deconvolution of peaks, and the black line corresponds to the sum of peaks fits (envelope).

Figure 5 shows the deconvolution of O 1s and Sn 3d XPS core level spectra recorded on the films. The O 1s core level peak exhibits slight asymmetry with a distinct shoulder in all the investigated samples suggesting the presence of three components (Figure 5). The main component centered at ~530.3 eV is assigned to lattice oxygen (O_L_) (oxygen directly bounded to a metal atom) [32]. The two other components, found at higher binding energy ~531.6 and ~532.4 eV, are connected with the oxygen vacancies (O_V_) mandatory for charge compensation after doping and the chemosorbed oxygen (O_Chem_), respectively, as in the previous literature [33].

The Sn 3d region of the three samples (Figure 5) presents doublets at 486.5 and 494.9 eV for Sn 3d_5/2_ and Sn 3d_3/2_, respectively. According to the literature [32], the three oxidation states of Sn atoms are defined by three binding energy values as the following Sn^0^ (485.0 eV), Sn^2+^ (485.9 eV), and Sn^4+^ (486.6 eV). Our results indicate the absence of Sn^0^ and Sn^2+^ within the films and therefore confirm the Sn^4+^ oxidation state and the formation of SnO_2_ by spray pyrolysis.

**Figure 6:**
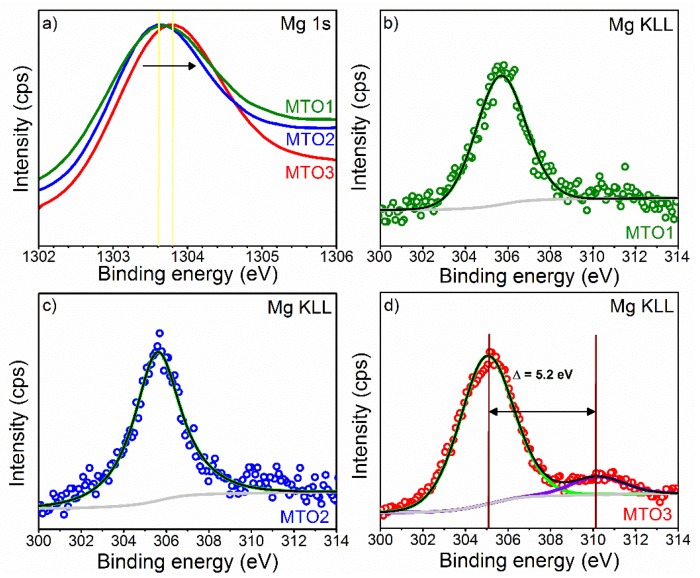
XPS of (**a**) Mg 1s core level spectra and ((**b**)−(**d**)) Mg KLL Auger spectra at MTO1, MTO2, and MTO3. The circles denote experimental data, colored lines demonstrate the deconvolution of peaks, and the black line corresponds to the sum of peaks fits (envelope).

XPS results also show the Mg 1s core level peak and the Mg KLL Auger emission peak. Estimation of the content of Mg at the samples indicate lower contents for MOT1 and MTO2 (~0.7 at.%) respect to MTO3 (~1 at.%). The results in Figure 6a show the Mg 1s spectrum for the MTO1 and MTO2 films at a lower energy (~1303.6 eV) than that recorded for the MTO3 films, which display a shift to a higher energy (~1303.8 eV) most likely due to the oxidation of Mg [30,34]. The Mg KLL Auger spectrum (Figure 6b–d) supports this fact by showing a component associated to Mg metal in the three samples and a second component (only present in the MTO3 films and shifted 5.2 eV to higher energy) assigned to Mg oxide [35,36]. These results are in line with the MgO diffractions identified by XRD (Figure 2). 

Overall, the analysis of the material demonstrates the formation of Mg-doped SnO_2_. These results indicate that the morphological differences in the films (i.e., MTO1, MTO2, and MTO3) are attributed to the variation of Mg doping level in the SnO_2_ films. The incorporation of the Mg precursor in the spray solution affects the nucleation process and growth conditions of the films. This also slightly deviates the crystalline structure of the doped films (with respect to pristine SnO_2_) and, therefore, the film morphology. As the doping reaches a saturation point, the alloy system tends to dissociate and a second phase material emerges (i.e., MgO) causing further change in the growth mechanism and the film morphology. Previously, other authors also observed morphological changes in SnO_2_ and ZnO films due to Eu (europium) [37] and Mg doping [38], respectively, and the formation of a MgO second phase material. 

### 3.2. Gas Sensing Properties

**Figure 7:**
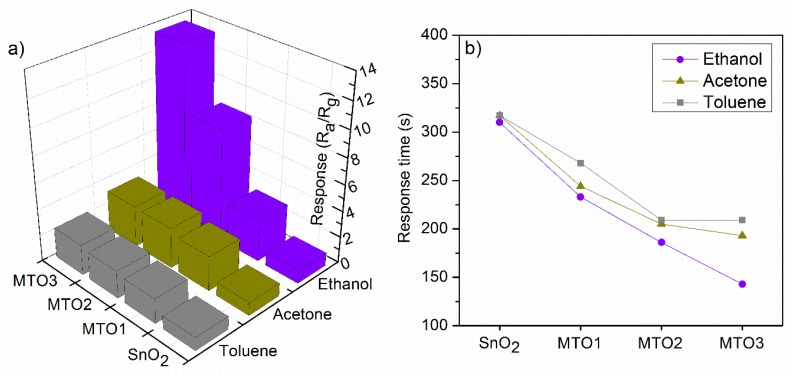
Response (**a**) and response time (**b**) towards 80 ppm of ethanol, acetone, and toluene for the non-doped and Mg-doped SnO_2_ films.

**Figure 8:**
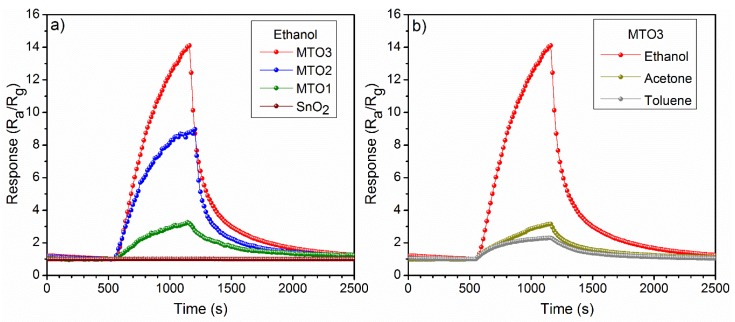
Response of the (**a**) non-doped and Mg-doped SnO_2_ films to 80 ppm of ethanol and (**b**) MTO3 response to 80 ppm of toluene, acetone, and ethanol recorded at 160 °C.

Non-doped and Mg-doped SnO_2_ films were exposed consecutively to various reductive gases (ethanol, acetone, and toluene) in a concentration of 80 ppm at an operating temperature (T_op_) of 160 °C. A summary of the response to each analyte and each sample is presented in Figure 7a. These results reveal low responses for the non-doped SnO_2_ film compared to the Mg-doped SnO_2_, which show higher responses to the target analytes as the Mg percentage increases in the films (e.g., MTO3 film response to ethanol is almost fourteen times higher than SnO_2_ film). In contrast, the response time (Figure 7b) shows a decreasing trend as the Mg percentage increases in the films. Generally, the electrical measurements also revealed an increase of the film electrical resistance by more than five orders of magnitude as the Mg percentage increases from 0 at.% to 1.6 at.%. In addition, the dynamic response to ethanol (Figure 8a) displayed a more reproducible and stable signal for the Mg-doped films compared to the non-doped film, particularly for those doped with a higher Mg concentration (MTO3). Similarly, the dynamic response to acetone and toluene showed reproducible and stable signals as shown in (Figure 8b) for the MTO3 samples. The results in Figure 7 and Figure 8b also show better responses towards ethanol than to acetone and toluene, in proportion to the increase of Mg doping. For instance, the MTO3 film responses were 13.5, 3.1, and 2.3 for a concentration of 80 ppm of ethanol, acetone, and toluene, respectively.

**Figure 9:**
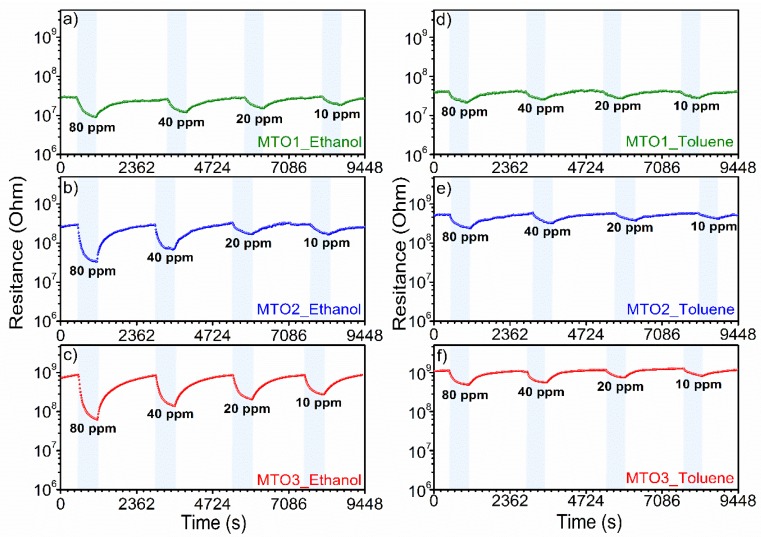
Dynamic response curves of Mg-doped SnO_2_ films at different concentrations of ethanol (**left figure**) and toluene (**right figure**).

**Figure 10:**
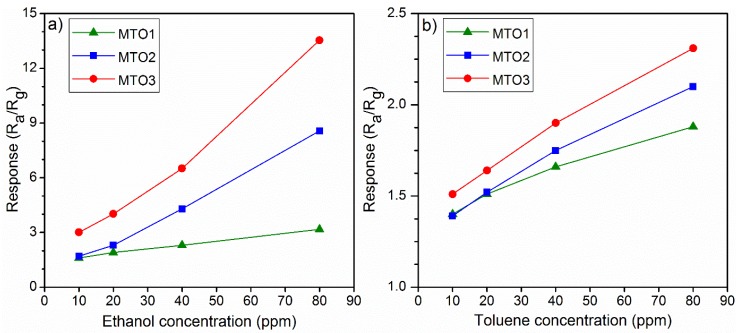
Response of Mg-doped SnO_2_ films vs. (**a**) ethanol and (**b**) toluene concentration at 160 °C.

Further testing of the Mg-doped SnO_2_ films to various concentrations of ethanol (highest response) and toluene (lowest response) from 10 ppm to 80 ppm displayed a proportional increase of the response with the gas concentration (Figure 9 and Figure 10). We also observe that by increasing the Mg percentage from 0.8 at.% (MTO1) to 1.6 at.% (MTO3), the response to ethanol increases by two times (from 1.6 to 3) for 10 ppm and by four times (from 3.2 to 13.5) for 80 ppm. Coincidently, we note that the response to ethanol obtained from the MTO1 film to 80 ppm is almost equal to the response obtained from the MTO3 film to 10 ppm. The same behavior, although with lower enhancement than that observed for ethanol, was observed for toluene. Hence, these results demonstrate a major enhancement of the response with the increase of Mg doping in SnO_2_ film, especially towards ethanol.

**Figure 11:**
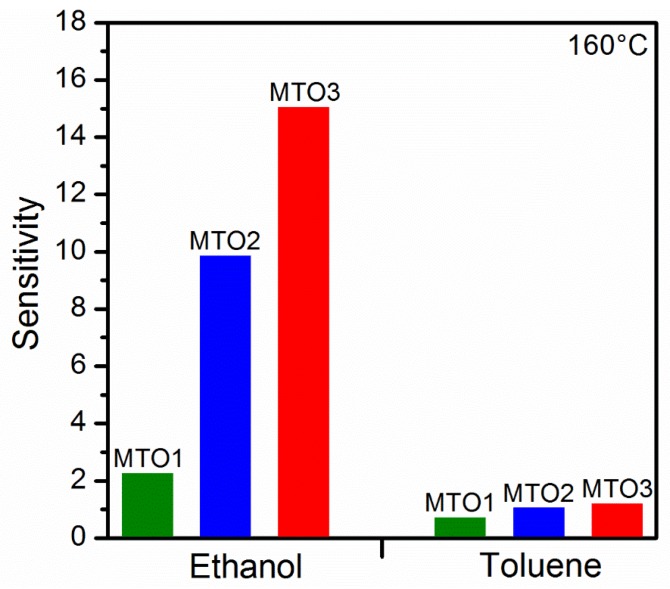
Sensitivity of the Mg-doped SnO_2_ films to ethanol and toluene.

Similarly, the film doping level also influences the sensitivity of the films (Figure 11), which could be favorable for tuning of the cross-sensitivity (ΔS). Thus, we observed lower cross-sensitivity and in turn, better selectivity between ethanol and toluene for the samples with higher doping levels (ΔS = 8.8 for MTO2 and ΔS = 13.9 for MTO3) as compared to the sample with low doping concentration (ΔS = 1.6 for MTO1). Thus, the results suggest partial selectivity to ethanol, particularly for the MTO3 films. 

**Figure 12:**
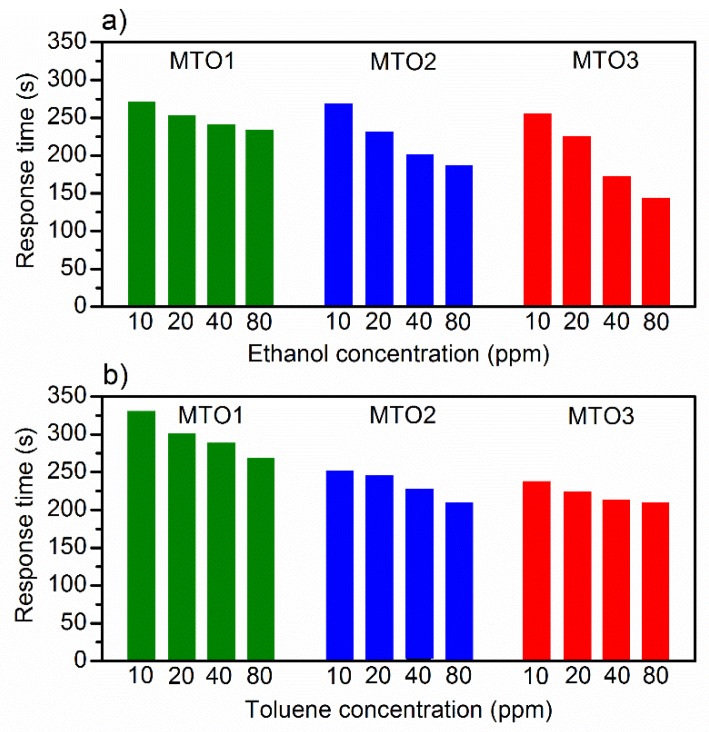
Response time to various concentrations of (**a**) ethanol, and (**b**) toluene.

Further analysis of the dynamic response of the films (Figure 9) shows that the response time (Figure 12) for ethanol and toluene is inversely related to the gas concentration in both cases. For instance, for ethanol at 80 ppm, the response time decreases from 233 s (MTO1) to 143 s (MTO3), which is 90 s faster, by doubling the Mg percentage in the SnO_2_ film (from 0.8 at.% to 1.6 at.%). Similar behavior is observed for toluene with a response time decrease of 59 s for MTO3 with respect to MTO1. The results also show a complete recovery of the base line in the three systems, which is accelerated by increasing the Mg percentage in the films (Figure 9). 

Overall, the films displayed a low drift of the baseline resistance during each test, most likely connected with the low operating temperature. Despite this, the response magnitudes did not show significant changes. Generally, the MTO3 films showed better medium-term stability displaying 8% and 6% less deviation in the base line resistance during the whole testing period (see experimental section) compared to the MTO1 and MTO2 films, respectively. Further analysis after the testing period and after having exposed the films to all target analytes showed no significant changes in the main core levels XPS spectrum of tin, magnesium, and oxygen, supporting the stability of the film. 

Table 2 presents a comparison of our results and those for other materials in the literature. The table summarizes the material synthesis method, testing conditions (concentration, operating temperature), and response reported to ethanol and toluene. In this summary, one can notice the use of various materials for sensing both gases, including intrinsic oxides (e.g., SnO_2_, WO_3_, Fe_2_O_3_) and modified (e.g., SnO_2_/ZnO, SnO_2_/MgAl_2_O_4_) or doped (Mg-doped ZnO, Pr-doped SnO_2_) oxides. Despite the comparison of the data being complex, as gas sensing results depend on not only the sensing material but also the test conditions, we consider these data still meaningful to offer a general idea of the properties of our samples. Overall, we observe that our results are in agreement with the literature and that the responses recorded to ethanol and toluene with our samples are higher or in the same order at a comparatively lower operating temperature (160 °C) and lower concentration (80 ppm) than other works (which show operating temperatures above 200 °C and typically concentrations of 100 ppm). The response time recorded for the works in the literature is in general in the same order of magnitude (hundreds of seconds) as for our films. Table 2 also includes an example of a similar system (i.e., Mg-doped SnO_2_ via spray pyrolysis) although tested towards a different analyte (LPG—liquid petroleum gas). The response (1.4) of this system to 1000 ppm of LPG was registered at 285 °C. 

### 3.3. Gas Sensing Mechanism

**Figure 13:**
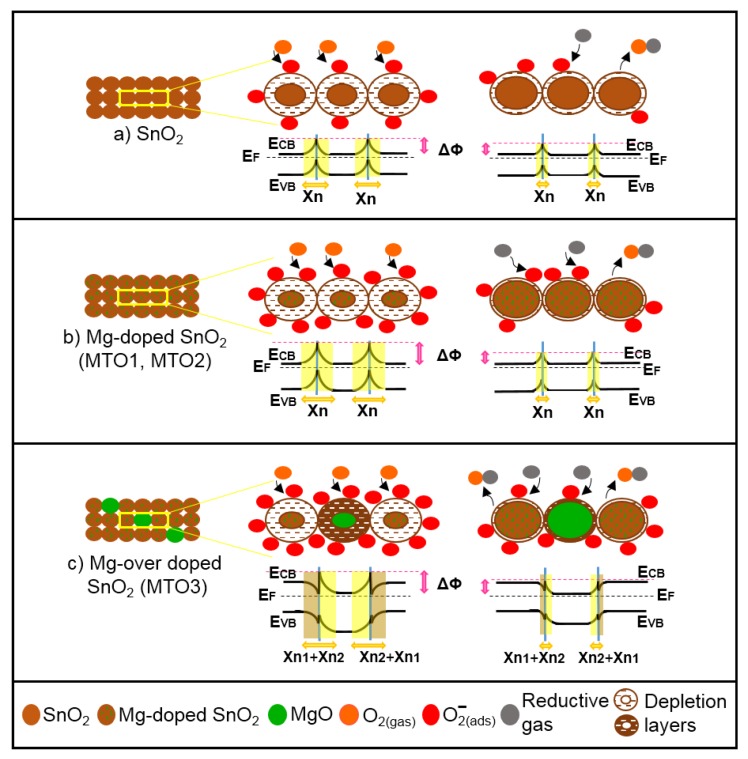
Schematic representation of the gas sensing mechanisms of (**a**) non-doped and (**b** and **c**) Mg-doped SnO_2_ films in air (left) and reductive gas (right). E_CB_ is the bottom of conduction band; E_F_ is the bulk Fermi level; E_VB_ is the top of valence band; ΔΦ is the built-in potential barrier; Χn, and Χn_2_ are depth of the depletion layer from the surface; Χn_1_ is the depth of the accumulation layer from the surface (not to scale).

Generally, the gas sensing results showed that all films behave as typical n-type semiconductors when exposed to gases, i.e., decreasing the overall electrical resistance in the presence of reducing gases such as ethanol, acetone, and toluene. This is consistence with the most accepted sensing mechanism model proposed in the literature for pristine metal oxides including SnO_2_ [46]. According to this model (Figure 13a), when SnO_2_ film is exposed to air, oxygen molecules are adsorbed at the surface and form oxygen ions [47,48] (in our case O_2_^−^ is formed because the operating temperature is 160 °C [11]), thus extracting electrons from the conduction band. As a result of this electron transfer, a depletion layer is formed and the film electrical resistance is increased. As soon as SnO_2_ film is exposed to a desired concentration of a reducing gas, such as ethanol, acetone or toluene, the reductive gas molecules will interact with oxygen negative ions and transfer the electrons back to the SnO_2_ conductive band. Therefore, the depletion layer narrows and the film electrical resistance decreases [46].

This mechanism is similar for the Mg-doped films (Figure 13b). However, in this system the incorporation of Mg into the SnO_2_ structure enhances the sensing mechanism due to the lattice disorder introduced by the substitution of Sn^4+^ ions by Mg^2+^ ions and the subsequent diminution of the crystallite size by nearly three times with respect to the pristine SnO_2_ films (Table 1). Then, more reactive oxygen molecules are adsorbed in the pre-adsorption cycle and available to interact with the reductive gases [16,49]. 

The mechanism for the over-doped films (Figure 13c), however, includes a new component, introduced by the second phase particles (i.e., MgO) rather than by the change in crystalline size (notice the crystallite size of MTO1, MTO2, and MTO3 is similar). The presence of second phase particles with low loadings enhances further the sensing mechanism via a ‘spillover effect’ [50], which can be connected with the faster response and recovery time of MTO3 with respect to MTO2 and MTO1. The second phase particles also introduce an extra potential barrier formed between the MgO and Mg-doped SnO_2_ (heterojunction). This extra potential barrier may accumulate or deplete extra-pre-adsorbed oxygen in the boundary grain to grain, thus maximizing the electrical resistance change in the gas (analyte) adsorption stage, as noticed earlier for other gas sensitive materials with nanoscaled heterojunctions [6,51]. 

On the other hand, the visibly different morphologies in the films, as result of the Mg doping, could also play a role in the sensing performance by providing a different electrical percolation and possible fluctuations (noise) in the grain boundaries. Although the current dc measurements do not give evidence of the level of these fluctuations, we cannot rule out the presence of a parallel mechanism dependent on the grain boundaries, as noticed previously for ethanol and hydrogen by low-frequency noise measurements [52].

## 4. Conclusions

Mg-doped SnO_2_ thin films synthesized via a spray pyrolysis method were investigated for ethanol, acetone, and toluene sensing. Overall, the Mg-doped SnO_2_ films proved a more reproducible sensing behavior with complete recovery of the base line resistance compared to the non-doped SnO_2_ films. This paper proposes that increasing the Mg-doping level (from 0.8 at.% to 1.6 at.%) is responsible for the improvement of the sensing properties due to the decrease in the crystallites’ size and to the increase in defects in the SnO_2_ films. The results indicate that this remarkable improvement is also connected with the formation of MgO, which favors the spillover effects at the film and incorporates extra potential barriers formed between the MgO and the Mg-doped SnO_2_ grains. Both components (i.e., size reduction and the incorporation of MgO) play an important role in enhancing the ethanol sensing behavior of SnO_2_, and thus, the response of the 1.6 at.% Mg-doped SnO_2_ films is four times higher and 90 s faster than the response of the 0.8 at.% Mg-doped SnO_2_ films.

## Figures and Tables

**Table 1:** Lattice parameters and average crystalline size of the Mg-doped SnO_2_ thin films.

Sample	Unit Cell Parameters			Unit Cell Volume	Average Crystallite Size
	*a*=*b* (Å)	*c* (Å)	*α = β = γ*	V = *a*^2^*c* (Å^3^)	D (nm)
SnO_2_	4.732	3.187	90°	71.39	26 *
MTO1	4.739	3.187	90°	71.58	9.8
MTO2	4.742	3.181	90°	71.51	9.6
MTO3	4.742	3.181	90°	71.51	9.5

* SnO_2_ average crystallite size was estimated in our previous work [27].

**Table 2:** Summary of the materials, testing conditions and responses to ethanol and toluene reported in the literature and in our work for MTO3.

Material	Method	Morphology	Concentration (ppm)	T_op_ (°C)	R_a_/R_g_	t_res_ (s)	Ref
**Mg-doped SnO_2_**	**Spray pyrolysis**	**Nanospheres**	**Ethanol** **80**	**160**	**13.5**	**143**	**This work**
SnO_2_/ MgAl_2_O_4_	solid-state and sol-gel	Nanocomposites	Ethanol 100	227	3.33 *	--	[39]
Au/Mg-TiO_2_/SnO_2_	Hydrothermal	Heterostructure nanosheets	Ethanol 50	260	7	--	[40]
Mg-doped ZnO	RF magnetron sputtering	Rod-like	Ethanol 50	100	2.32 *	--	[19]
Ce-doped SnO_2_	Cosputtering	Nanostructures	Ethanol 100	225	5	4	[14]
Zn-doped SnO_2_	Hydrothermal	Hierarchical architectures	Ethanol 100	213	13.8	--	[16]
SnO_2_/ZnO	Hydrothermal	Nanostructures	Ethanol 100	400	6.2	--	[41]
Al-doped SnO_2_	Chemical synthesis	powders	Ethanol 100	280	35.25	--	[12]
Pr-doped SnO_2_	Electro-spinning	Hollow nanofibers	Ethanol 100	300	28.62	168	[15]
**Mg-doped SnO_2_**	**Spray pyrolysis**	**Nanospheres**	**Toluene** **80**	**160**	**2.3**	**209**	**This work**
SnO_2_	Hydrothermal	Flower microstructures	Toluene 10	250	1	--	[42]
Pt-doped SnO_2_	LPCVD	Island-like structures	Toluene 25	440	6	--	[13]
PdO-decorated ZnO	Hydrothermal	Flower nanostructures	Toluene 100	160	1.9	--	[7]
Fe_2_O_3_	Hydrothermal	Nanoshuttles	Toluene 100	440	2.3	5	[43]
Co_3_O_4_	Solvothermal	Nanorods	Toluene 200	120	3	--	[44]
WO_3_	Vapor phase synthesis	Nanoneedles	Toluene 100	250	2.2	398	[6]
Pt@WO_3_	Vapor phase synthesis	NPs@NNs	Toluene 100	250	7	100	[6]
Fe_2_O_3_@WO_3_	Vapor phase synthesis	NPs@NNs	Toluene 100	250	8	150	[6]
Mg-doped SnO_2_	Spray pyrolysis	Leaf-like grains	LPG 1000	285	1.4 *	--	[45]

T_op_: temperature of operation, t_res_: response time, * R_a_/R_g_ calculated from [19,39,45]
